# Exercise capacity-hemodynamics mismatch in elderly patients with pulmonary hypertension: A nationwide multicenter study from Taiwan Society of Cardiology Pulmonary Hypertension Registry (TAIPANS)

**DOI:** 10.1016/j.heliyon.2024.e27537

**Published:** 2024-03-11

**Authors:** Chang-Ying Chen, Wang Mei-Tzu, Shih-Hsien Sung, Yih-Jer Wu, Chih-Hsin Hsu, Wan-Jing Ho, Yen-Hung Lin, Wei-Shin Liu, Ju-Chi Liu, Yung-Ta Kao, Wen-Shiann Wu, Chun-Hsien Wu, Meng-Huan Lei, Yu-Wei Chen, Chien Chen-Yu, Yu-Wei Chiu, Zen-Kong Dai, Tsung-Hsien Lin, Lin Lin, Cheng-Chih Chung, Chang-Min Chung, Sung-Hao Huang, Chin-Chang Cheng, Yen-Wen Wu, Ting-Hsing Chao, Juey-Jen Hwang, Charles Jia-Yin Hou, Wei-Chun Huang

**Affiliations:** a Department of Critical Care Medicine, Kaohsiung Veterans General Hospital, Kaohsiung, Taiwan; b Department of Internal Medicine, National Cheng Kung University Hospital, College of Medicine, National Cheng Kung University, Tainan, Taiwan; c School of Medicine, National Yang-Ming Chiao-Tung University, Taipei, Taiwan; d Division of Cardiology, Department of Internal Medicine, Taipei Veterans General Hospital, Taipei City, Taiwan; e Department of Medicine, MacKay Medical College, New Taipei, Taiwan; f Cardiovascular Center, Department of Internal Medicine, MacKay Memorial Hospital, Taipei, Taiwan; g Department of Cardiology, Chang Gung Memorial Hospital, College of Medicine, Chang Gung University, Tao-Yuan, Taiwan; h Division of Cardiology, Department of Internal Medicine, National Taiwan University Hospital, Taipei City, Taiwan; i Division of Cardiology, Tzu-Chi General Hospital, Hualien, Taiwan; j Division of Cardiology, Department of Internal Medicine, Shuang Ho Hospital, Taipei Medical University, New Taipei City, Taiwan; k Taipei Heart Institute, Taipei Medical University, Taipei, Taiwan; l Division of Cardiology, Department of Internal Medicine, School of Medicine, College of Medicine, Taipei Medical University, Taipei, Taiwan; m Division of Cardiology, Department of Internal Medicine, Taipei Medical University Hospital, Taiwan; n Department of Cardiology, Chi-Mei Medical Center, Tainan, Taiwan; o Division of Cardiology, Department of Internal Medicine, Tri-Service General Hospital, National Defense Medical Center, Taipei, Taiwan; p Cardiovascular Center, Lo-Tung Poh-Ai Hospital, YI-Lan, Taiwan; q Cardiovascular Center, Taichung Veterans General Hospital, Taichung, Taiwan; r Institute of Clinical Medicine, National Yang Ming Chiao Tung University, Taipei, Taiwan; s Department of Post-Baccalaureate Medicine, College of Medicine, National Chung Hsing University, Taichung, Taiwan; t Department of Internal Medicine, Dalin Tzu Chi Hospital, Buddhist Tzu Chi Medical Foundation, Chiayi, Taiwan; u Division of Cardiology, Cardiovascular Medical Center, Far Eastern Memorial Hospital, New Taipei City, Taiwan; v Department of Computer Science and Engineering, Yuan Ze University, Taoyuan, Taiwan; w Department of Pediatrics, Kaohsiung Medical University Hospital, Kaohsiung, Taiwan; x Graduate Institute of Medicine, College of Medicine, Kaohsiung Medical University, Kaohsiung, Taiwan; y Department of Pediatrics, School of Medicine, College of Medicine, Kaohsiung Medical University, Kaohsiung, Taiwan; z Division of Cardiology, Department of Internal Medicine, Kaohsiung Medical University Hospital, Taiwan; aa Faculty of Medicine, Kaohsiung Medical University, Kaohsiung, Taiwan; ab Cardiovascular Center, National Taiwan University Hospital, Hsin-Chu Branch, Hsinchu, Taiwan; ac Division of Cardiovascular Medicine, Department of Internal Medicine, Wan Fang Hospital, Taipei Medical University, Taipei, Taiwan; ad Division of Cardiology, Department of Internal Medicine, Chiayi Chang Gung Medical Foundation, Chiayi County, Taiwan; ae Medical Department, Chiayi Chang Gung Medical Foundation, Chiayi County, Taiwan; af Division of Cardiology, National Yang Ming Chiao Tung University Hospital, Yi-Lan, Taiwan; ag Department of Physical Therapy, Fooyin University, Kaohsiung, Taiwan; ah Graduate Institute of Medicine, Yuan Ze University, Taoyuan, Taiwan; ai National Sun Yat-sen University, Kaohsiung, Taiwan

**Keywords:** Pulmonary hypertension, Elderly, Hemodynamic

## Abstract

**Background:**

Demographics of pulmonary hypertension (PH) has changed a lot over the past forty years. Several recent registries noted an increase in mean age of PH but only a few of them investigated the characteristics of elderly patients. Thus, we aimed to analyze the characteristics of PH in such a population in this study.

**Methods:**

This multicenter study enrolled patients diagnosed with PH in group 1, 3, 4, and 5 consecutively from January 1, 2019 to December 31, 2020. A total of 490 patients was included, and patients were divided into three groups by age (≤45 years, 45–65 years, and >65 years).

**Results:**

The mean age of PH patients diagnosed with PH was 55.3 ± 16.3 years of age. There was higher proportion of elderly patients classified as group 3 PH (≤45: 1.3, 45–65: 4.5, >65: 8.1 %; *p* = 0.0206) and group 4 PH (≤45: 8.4, 45–65: 14.5, >65: 31.6 %; *p* < 0.0001) than young patients. Elderly patients had shorter 6-min walking distance (6 MWD) (≤45 vs. >65, mean difference, 77.8 m [95% confidence interval (CI), 2.1–153.6 m]), lower mean pulmonary arterial pressure (mPAP) (≤45 vs. >65, mean difference, 10.8 mmHg [95% CI, 6.37–15.2 mmHg]), and higher pulmonary arterial wedge pressure (PAWP) (≤45 vs. 45–65, mean difference, −2.1 mmHg [95% CI, −3.9 to −0.3 mmHg]) compared to young patients. Elderly patients had a poorer exercise capacity despite lower mPAP level compared to young population, but they received combination therapy less frequently compared to young patients (triple therapy in group 1 PH, ≤45: 16.7, 45–65: 11.3, >65: 3.8 %; *p* = 0.0005). Age older than 65 years was an independent predictor of high mortality for PH patients.

**Conclusions:**

Elderly PH patients possess unique hemodynamic profiles and epidemiologic patterns. They had higher PAWP, lower mPAP, and received combination therapy less frequently. Moreover, ageing is a predictor of high mortality for PH patients. Exercise capacity-hemodynamics mismatch and inadequate treatment are noteworthy in the approach of elderly population with PH.

## Highlights

• Old patients were symptomatic at lower mean pulmonary artery pressure.
• Old patients less frequently receive combination therapy as young patients.
• Old patients were more commonly diagnosed with group 3 and 4 pulmonary hypertension.
• Age older than 65 years was a predictor for mortality of pulmonary hypertension.

## 1 Introduction

Pulmonary hypertension (PH) is high blood pressure in the pulmonary blood vessels. It results in damage to the right heart, leading to right heart failure and ultimately death if untreated. PH is divided into five groups on the basis of pathophysiology, clinical presentation, hemodynamic characteristics and therapeutic management. Based on European Society of Cardiology (ESC)/European Respiratory Society (ERS) PH Guidelines classification, group 1 is pulmonary arterial hypertension (PAH), group 2 is PH due to left heart disease, group 3 is PH due to lung diseases and/or hypoxia, group 4 is chronic thromboembolic pulmonary hypertension, and group 5 is PH with unclear and/or multifactorial mechanisms [1,2].

Many registries have been established to assess the characteristics of patients suffering from PH since 1980s. They demonstrated an increasing trend in the mean age of PH patients over the past forty years [3]. In the first registry of PH conducted by the National Institute of Health in the United States between 1981 and 1985, only 9% of patients were >60 years of age with a mean age of 36 years [4]. In 2012, Assessing the Spectrum of Pulmonary Hypertension Identified at a Referral Center (ASPIRE) registry enrolling 1344 cases of PH between 2001 and 2010 showed a mean age of 59 years [5].

Despite the fact that the mean age of patient with PH rose dramatically in the past half-century, rare large registries investigated the characteristics of PH in the elderly population. To our knowledge, there were only two large studies specifically investigating the characteristics of elderly patient with PH recently. One was the study of a PH registry in the United Kingdoms and Ireland in 2012 [6]. Another was the study analyzing the Comparative, Prospective Registry of Newly Initiated Therapies for Pulmonary Hypertension (COMPERA) registry in 2013 [7]. As a result, the understanding of the difference of baseline characteristics between young and old patients diagnosed with PH remained limited. Our objective is to examine the disparities in epidemiology, symptoms, exercise capacity, hemodynamic profiles, and treatment among patients with PH across various age groups in order to provide insight into the difference between young and elderly patients with PH.

## 2 Methods

### 2.1 Settings and participants

In January 2019, Taiwan Society of Cardiology Pulmonary Hypertension Registry (TAIPANS) was launched by Taiwan Society of Cardiology. This multicenter study analyzed data of TAIPANS, including demographics, hemodynamics, etiology, clinical presentations, clinical course, and management of patients diagnosed with PH. All consenting patients diagnosed with PH according to criteria at participating institutions were consecutively enrolled. All the patients were prevalent cases. Patients underwent clinical assessments and receive standard medical care as determined by the patient's physician. The intention of this registry was to gather data on PH patients under condition of routine clinical care. Therefore, patients did not receive experimental intervention or treatment as a consequence of their participation in the registry, and this registry did not provide a therapy protocol or prescribe a predetermined visit schedule. In addition, participating patients will be followed for a minimum of 5 years from the time of enrollment. The study protocol conforms to the ethical guidelines of the 1975 Declaration of Helsinki as reflected in a priori approval by the institution's human research committee.

This study analyzed data of the TAIPANS enrollment between January 1, 2019 and December 12, 2020, and there was no outcome reports due to study design. The inclusion criteria included that patient were alive and in age over 20 years at the time of enrollment. Patient or his/her legal representative should sign the informed consent approved by institutional ethics committee. Patient must be diagnosed as group 1, 3, 4, 5 based on 2015 ESC/ERS PH Guidelines classification [2]. Furthermore, all PH diagnosis must be confirmed by right heart catheterization (RHC) and achieved mPAP >20 mmHg at rest, PAWP <15 mmHg, and pulmonary vascular resistance (PVR) > 3 Wood units. The patients would be excluded if they were diagnosed as pulmonary veno-occlusive disease, pulmonary capillary hemangiomatosis or group 2 PH. Another study enrolling patients with pulmonary hypertension related to left heart diseases and a study enrolling patents with group 4 PH receiving pulmonary endarterectomy (PEA) are currently underway, and the exclusion of patients group 2 PH and group 4 PH who had received pulmonary endarterectomy were implemented to prevent duplicate enrollment. We divided the patient populations into young-aged group with age ≤65 years, middle-aged group with age between 45 and 65 years, and elderly group with age >65 years in order to examine the trend of characteristic change of PH patients depending on age.

Multiparameter of clinical signs were collected, including sex, body mass index (BMI), clinical signs of right heart failure (HF), syncope, progression of symptom (categorized into no progression, slow progression, and rapid progression of symptoms according to 2015 ESC/ERS Guidelines for the diagnosis and treatment of pulmonary hypertension [8]), Word Health Organization functional (WHO Fc) class; hemodynamic profile, including right atrium (RA) pressure, mean pulmonary arterial pressure (mPAP), pulmonary arterial wedge pressure (PAWP), cardiac index, PVR, and mixed venous oxygen saturation (SvO2) were recorded; cardiopulmonary function and exercise testing were contained, including 6-min walking distance (6 MWD), pulmonary function including total lung function (TLC), forced expiratory volume during the first second (FEV1), forced vital capacity (FVC), and FEV1/FVC, peak oxygen consumption (VO2), percentage of predicted VO2, minute ventilation/carbon dioxide production (VE/VCO2); biomarker and images were acquired, including B-type natriuretic peptide (BNP), echocardiography and cardiovascular magnetic resonance (CMR) measuring left ventricle ejection fraction (LVEF), right ventricle ejection fraction (RVEF), RA area, and pericardial effusion; medication therapies were documented, including phosphodiesterase-5 inhibitors (PDE-5i), endothelium receptor antagonists (ERA), and prostacyclin analogues. The proportion of patients with group 4 PH receiving balloon pulmonary angioplasty (BPA) was recorded.

### 2.2 Statistical analysis

All data were processed using SAS software (version 9.4; SAS Institute Inc., Cary, NC). A *p*-value ≤0.05 was considered statistically significant throughout the study.

Data was presented as the mean ± standard deviation (SD) for continuous variables, and as number and percentages (%) for categorical variables. The groups were compared using the one-way analysis of variance (ANOVA) with post-hoc Scheffé testing for numerical data after verifying the equality of variances. Pearson's chi-squared test with post-hoc pairwise Z-tests was used for analyzing categorical data (*p* value was adjusted with Bonferroni method). Correlation between mPAP and age and correlation between 6 MWD and age were assessed with Pearson correlation coefficients. Multivariate logistic regression was used to identify the risk factors associated with high mortality risk in pulmonary hypertension. The odds ratios (OR) and their associated 95% CI for each potential risk factor were presented. The determination of mortality risk of PH patients was based on the risk stratification model proposed by 2015 ESC/ERS guidelines for the diagnosis and treatment of pulmonary hypertension [2]. Due to the lack of a recognized risk stratification model for other PH groups, we have adopted the PAH risk stratification model to assess the risk factors for high risk in the overall pulmonary hypertension population.

## 3 Results

### 3.1 Patients characteristics at time of enrollment

**Fig. 1:**
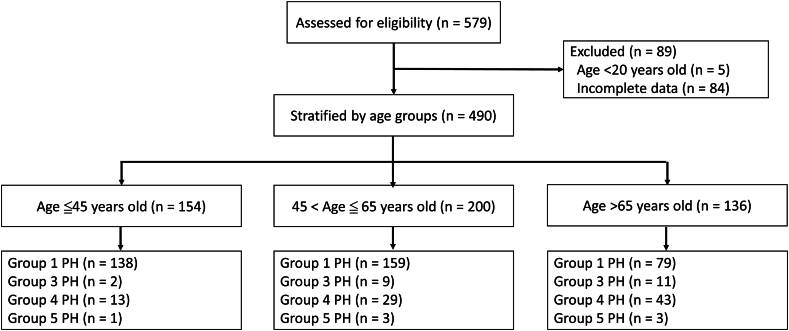
Study flow chart.

A total of 579 patients with pulmonary hypertension had been enrolled into the TAIPANS registry. 5 patients were excluded due to age <20 years old and 84 patients were not analyzed because of incomplete data (Fig. 1). Baseline characteristics of the patient ≤45 years, 45–65 years, and >65 years were shown in Table 1. The mean age was 55.3 (SD = 16.3 years), and there was 19.1% of patients older than 65 years of age. The median (interquartile range) of the time since diagnosis in different age groups were ≤45: 960 (1569), 45–65: 1062 (1661), >65: 730 (1343) days. There was no statistically significant difference in sex ratio across the three age groups (male in ≤45: 24.1, 45–65: 23.1, >65: 19.1 %; *p* = 0.6981).

**Table 1:** Basic characteristics for patients in Taiwan Society of Cardiology Pulmonary Hypertension Registry (TAIPANS).

	Age ≤45 years (n = 154)	Age 45–65 years (n = 200)	Age >65 years (n = 136)	*p*-Value
Different etiology for PH, n (%)				
Group 1	138 (89.6) *^,^ ‡	159 (79.5) †, ‡	79 (58.1) *,†	<0.0001
Group 3	2 (1.3) *	9 (4.5)	11 (8.1) *	0.0206
Group 4	13 (8.4) *	29 (14.5) †	43 (31.6) *,†	<0.0001
Group 5	1 (0.6)	3 (1.5)	3 (2.2)	0.534
Male, n (%)	42 (24.1)	55 (23.1)	31 (19.1)	0.6981
Body mass index, kg/m^2^	24.4 ± 5.5	24.4 ± 5.4	24.6 ± 4.8	0.9503
Clinical signs of right heart failure, n (%)	35 (28.0)	57 (33.0)	30 (24.2)	0.2509
Syncope, n (%)				0.2601
No syncope	154 (88.5)	221 (92.9)	154 (95.1)	
Occasional	15 (8.6)	13 (5.5)	6 (3.7)	
Repeated	5 (2.9)	4 (1.7)	2 (1.2)	
Progression of symptoms, n (%)				0.6605
No progression	76 (43.7)	106 (44.5)	69 (42.6)	
Slow	82 (47.1)	101 (42.4)	77 (47.5)	
Rapid	16 (9.2)	31 (13.0)	16 (9.9)	
Six-minute walking distance, m	367.2 ± 97.3 *	360.2 ± 320.8	289.3 ± 110.7 *	0.0235
BNP, ng/L	316.3 ± 492.5	326.7 ± 483.0	356.9 ± 433.6	0.8829
Pulmonary function				
Total lung capacity, L	4.9 ± 1.6 *^,^ †	4.1 ± 1.1 †	3.7 ± 1.0 *	0.0003
FEV1, L	2.4 ± 0.8	3.9 ± 14.3	1.4 ± 0.5	0.1270
FVC, L	3.5 ± 5.3	3.5 ± 10.3	1.9 ± 0.7	0.1993
FEV1/FVC, %	83.2 ± 8.3 *^,^ †	77.9 ± 15.4 †	78.2 ± 11.9 *	0.0053
DLCO, %	57.6 ± 24.7	51.0 ± 25.2	50.3 ± 30.3	0.2164
Hemodynamics				
Right atrium pressure, mmHg	11.6 ± 9.6	12.1 ± 7.6	11.5 ± 12.6	0.8868
mPAP, mmHg	49.2 ± 13.8 *^,^ †	45.2 ± 14.6 †^,^ ‡	38.4 ± 14.4 *^,^ ‡	<0.0001
PAWP, mmHg	10.1 ± 4.1	12.0 ± 5.3	12.1 ± 6.0	0.0338
Cardiac index, L∙min^−1^∙m^−2^	2.6 ± 0.9	2.7 ± 1.0	3.0 ± 3.1	0.2382
PVR, Wood units	12.1 ± 11.3	11.5 ± 14.5	10.1 ± 14.2	0.5337
SvO2, %	62.0 ± 9.9	62.5 ± 11.8	58.8 ± 14.5	0.0953
Cardiopulmonary exercise testing				
Peak VO2, mL∙min^−1^∙kg^−1^	13.3 ± 4.5	12.3 ± 4.0	10.7 ± 3.6	0.1524
Percentage of predicted VO2, %	41 ± 12 *	51 ± 15	62 ± 24 *	0.0010
VE/VCO2, slope	46.1 ± 17.1	42.3 ± 15.7	39.6 ± 12.7	0.3548
Imaging (echocardiography or CMR)				
Left ventricle ejection fraction, %	65.2 ± 8.9	64.5 ± 8.1	62.7 ± 7.5	0.0538
Right ventricle ejection fraction, %	44.4 ± 29.6	38.1 ± 14.9	41.2 ± 13.9	0.5380
Right atrium area, cm^2^	21.7 ± 10.1	21.4 ± 8.2)	22.7 ± 8.1	0.8510
Pericardial effusion, n (%)	12 (6.9)	11 (4.6)	7 (4.3)	0.7129
Treatment, n (%)				
Phosphodiesterase-5 inhibitors	123 (70.7)	154 (64.7)	104 (64.2)	0.3512
Endothelium receptor antagonists	81 (46.6)	91 (38.2)	37 (22.8)	<0.0001
Prostacyclin analogues	24 (13.8)	25 (10.5)	5 (3.1)	0.0027
Balloon pulmonary angioplasty^a^, n (%)	8 (61.5)	11 (37.9)	19 (44.2)	0.3618

BNP, B-type natriuretic peptide; CMR, cardiovascular magnetic resonance; DLCO, diffusing capacity of the lung for carbon monoxide; FEV1, forced expiratory volume during the first second; FVC, forced vital capacity; IQR, interquartile range; mPAP, mean pulmonary artery pressure; PAWP, pulmonary artery wedge pressure; PH, pulmonary hypertension; PVR, pulmonary vascular resistance; SvO2, mixed venous oxygen saturation; VE/VCO2, minute ventilation/carbon dioxide production; VO2, peak oxygen consumption. Data are presented as median (IQR) for time since diagnosis, means ± SD for continuous variables, and as number and percentages (%) for categorical variables.*, †, ‡ indicates that post hoc Scheffé test or post-hoc pairwise Z-tests found a statistically significant difference between the marked groups a, the numbers inside the parentheses represent the proportion of patients receiving balloon pulmonary angioplasty or pulmonary endarterectomy in group 4 PH.

**Fig. 2:**
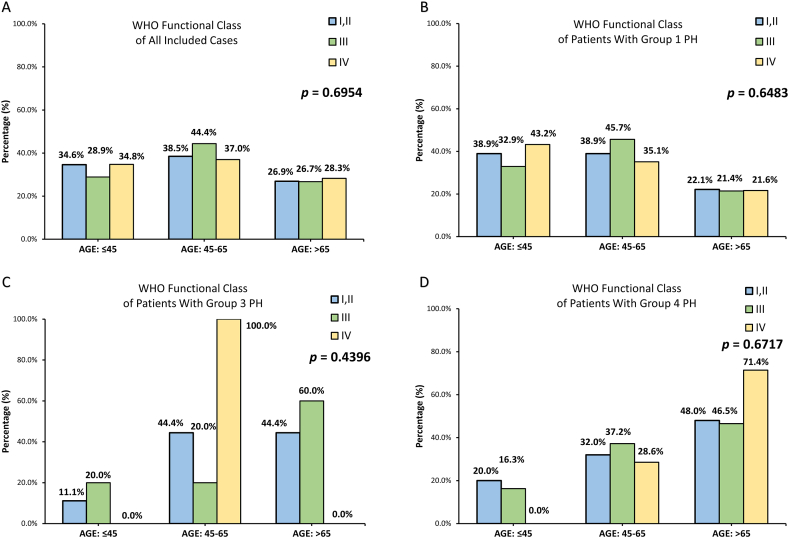
Distribution of World Health Organization (WHO) functional class (Fc) I/II, III, or IV across age groups within different pulmonary hypertension (PH) groups.

The proportion of clinical signs of right heart failure were similar across different age groups (≤45: 28.0, 45–65: 33.0, >65: 24.2 %; *p* = 0.2509). The frequency of syncope was also comparable between three age groups (no syncope: ≤45: 88.5, 45–65: 92.9, >65: 95.1 %; occasional syncope: ≤45: 8.6, 45–65: 5.5, >65: 3.7 %; repeated syncope: ≤45: 2.9, 45–65: 1.7, >65: 1.2 %; *p* = 0.2601). Moreover, there was no significant difference in the presence of rapid progression of symptoms across different age groups (rapid progression: ≤45: 9.2, 45–65: 13.0, >65: 9.9 %; *p* = 0.6605). Elderly patients had significantly shorter 6 MWD than young patients (≤45: 367.2 ± 97.3, 45–65: 360.2 ± 320.8, >65: 289.3 ± 110.7 m; *p* = 0.0235). Post hoc Scheffé test showed that the mean difference and 95% CI of 6 MWD between ≤45 and > 65 age groups were 77.8 m [2.1–153.6 m]. BNP levels were similar between three age groups (≤45: 367.2 ± 97.3, 45–65: 360.2 ± 320.8, 289.3 ± 110.7 m; *p* = 0.8829). Fig. 2A–D showed that distribution of WHO Fc I/II, III, or IV across age groups within each PH group were similar (Pearson's chi-square test, *p* = 0.6954 for all included cases, *p* = 0.6483 for patients with group 1 PH, *p* = 0.4396 for patients with group 3 PH, *p* = 0.6717 for patients with group 4 PH).

The distribution of WHO functional class did not differ significantly across age groups no matter in A, all included all included case (*p* = 0.6954); B, group 1 PH (*p* = 0.6483); C, group 3 PH (*p* = 0.4396); or D, group 4 PH (*p* = 0.6717). Data of patients with group 5 PH is not presented due to the rarity of cases. Pearson's chi-square test was used to assess whether there is significantly different distribution of WHO functional class across age groups within different PH groups.

Hemodynamic profile (Table 1) demonstrated that mPAP was lower (≤45: 49.2 ± 13.8, 45–65: 45.2 ± 14.6, >65: 38.4 ± 14.4 mmHg; *p* = <0.0001) and PAWP was higher (≤45: 10.1 ± 4.1, 45–65: 12.0 ± 5.3, >65: 12.1 ± 6.0 mmHg; *p* = 0.0338) in the elderly patient population. Post hoc Scheffé test showed that the mean difference and 95% CI of mPAP between ≤45 and 45–65 age groups were 4.1 mmHg [0.1–8.0 mmHg] and those between 45 and 65 and > 65 age groups were 6.7 mmHg [2.6 to 10.9]. The mean difference and 95% CI of PAWP evaluated by post hoc Scheffé test between ≤45 and 45–65 age groups were −2.1 mmHg [−3.9 to −0.3 mmHg]. In contrast, there was no significant difference in cardiac index (≤45: 2.6 ± 0.9, 45–65: 2.7 ± 1.0, >65: 3.0 ± 3.1 L min^−1^∙m^−2^; *p* = 0.2382) or PVR (≤45: 12.1 ± 11.3, 45–65: 11.5 ± 14.5, >65: 10.1 ± 14.2 wood units; *p* = 0.5337). Cardiopulmonary exercise testing including peak VO2 (≤45: 13.3 ± 4.5, 45–65: 12.3 ± 4.0, >65: 10.7 ± 3.6 mL min∙kg^−1^; *p* = 0.1524) and VE/VCO2 (≤45: 46.1 ± 17.1, 45–65: 42.3 ± 15.7), >65: 39.6 ± 12.7 %; *p* = 0.3548) also had comparable results across different age group. A better percentage of peak VO2 in elderly patients compared to young patients was noted (≤45 vs. >65, mean difference, −21.0% [95% confidence interval, −34.4 to −7.5%]; post hoc Scheffé test). In addition, LVEF (≤45: 65.2 ± 8.9, 45–65: 64.5 ± 8.1, >65: 62.7 ± 7.5 %; *p* = 0.0538) and RVEF (≤45: 44.4 ± 29.6, 45–65: 38.1 ± 14.9, >65: 41.2 ± 13.9 %; *p* = 0.5380) measured by echocardiography and CMR were similar between three age groups.

### 3.2 Etiology and treatment for PH

**Fig. 3:**
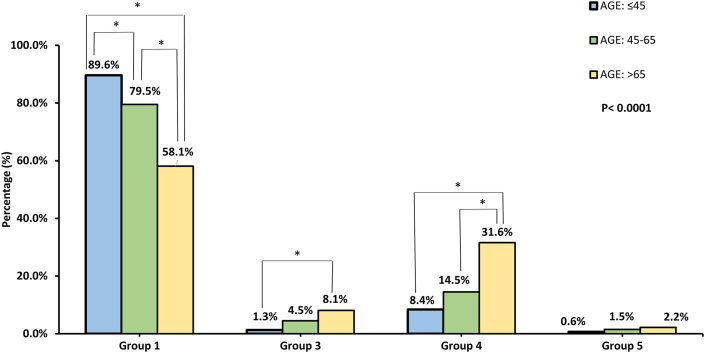
**Percentage of each age group in certain etiology of pulmonary hypertension (PH)**.

A significantly higher proportion of group 1 PH was reported in patient ≤45 years compared to other two age groups (≤45: 89.6, 45–65: 79.5, >65: 58.1 %; *p <* 0.0001; Fig. 3), and remarkably higher proportions of group 3 (≤45: 1.3, 45–65: 4.5, >65: 8.1 %; *p =* 0.0206; Fig. 3) and group 4 (≤45: 8.4, 45–65: 14.5, >65: 31.6 %; *p* < 0.0001; Fig. 3) PH were shown in elderly patients compared to the other two age groups. The exact number of patients in each group of PH across different age group was presented in Table 1.

**Fig. 4:**
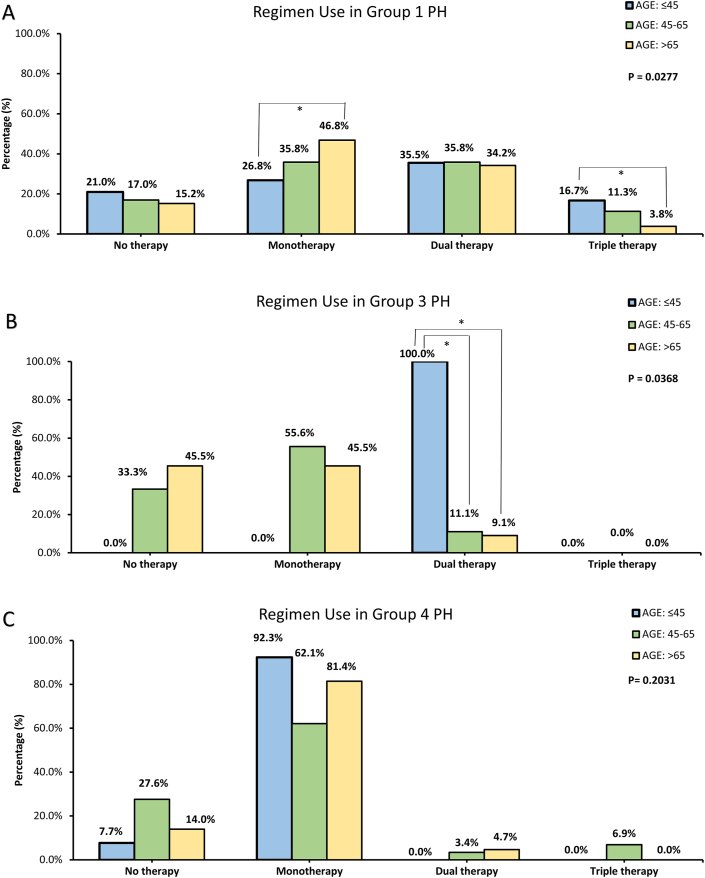
Percentage of each age group in certain regimen across different etiology of pulmonary hypertension (PH).

Significantly lower proportion of endothelium receptor antagonist (≤45: 46.6, 45–65: 38.2, >65: 22.8 %; *p* < 0.0001) and prostacyclin analogue (≤45: 13.8, 45–65: 10.5, >65: 3.1 %; *p* = 0.0027) were prescribed in elderly patients compared to young patients (Table 1). In group 4 PH, the proportion of patients receiving BPA was similar between different age groups (≤45: 61.5, 45–65: 37.9, >65: 44.2 %; *p* = 0.3618; Table 1). In group 1 PH, more young patients received triple therapy than elderly patient (triple therapy: ≤45: 16.7, 45–65: 11.3, >65: 3.8 %; *p* = 0.0005; Fig. 4A). In contrast, elderly patients were more frequently administered monotherapy with only one PH drug compared with younger population (≤45: 26.8, 45–65: 35.8, >65: 46.8 %; *p* = 0.0028; Fig. 4A). In group 3 PH, there were more patients ≤45 years received off-label dual therapy (≤45:100.0, 45–65: 1.1, >65: 9.1 %; *p* = 0.0007; Fig. 4B). In group 4 PH, the proportion of patients receiving different regimen was not significantly different across age groups (*p* = 0.2031; Fig. 4C).

There was a statistically significant difference between relative frequencies of patients diagnosed with different group of PH in each age groups (Pearson's chi-square test, p < 0.0001). * indicated that the proportion of patients diagnosed with certain group of PH in the denoted age groups differed significantly from each other (significant level had been adjusted with Bonferroni method). The proportion of elderly patients with group 1 PH was significantly lower than that of young patients while the proportions of elderly patients with group 3 and 4 PH were significantly higher than those of young patients.

A. In group 1 PH, there was a statistically significant difference in percentage of each age group in patients receiving different regimens (Pearson's chi-square test, *p* = 0.0277). Elderly patients received triple therapy less frequently and received monotherapy more frequently compared to young patients. B. In group 3 PH, no medications were approved by the Taiwan Food and Drug Administration. However, we still observed significantly higher proportion of young patients receiving off-label dual therapy than the elderly patients (Pearson's chi-square test, *p* = 0.0368). C. In group 4 PH, there was no significant difference in percentage of each age group in patients receiving different regimens. Data of patients with group 5 PH is not presented due to the rarity of cases. * indicated that the proportion of patients diagnosed with certain group of PH in the denoted age groups differed significantly from each other (significant level had been adjusted with Bonferroni method).

### 3.3 Correlation between age and mPAP/6 MWD and risk factors for high mortality risk of PH

**Fig. 5:**
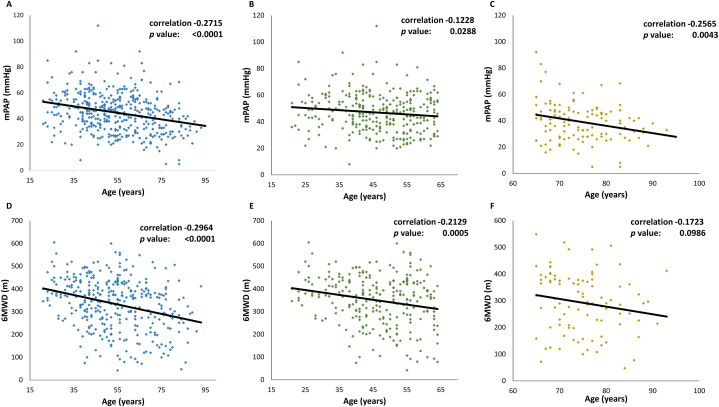
Correlation between age and mean pulmonary arterial pressure (mPAP) and 6-min walking distance (6 MWD). A. Correlation between age and mPAP. The diagram showed a linear trend towards lower mPAP with increasing age (r = −0.2715, p < 0.0001). B. The correlation between age and mPAP became less prominent (r = −0.1228; p = 0.0288) after excluding patients older than 65 years. C. Correlation between age and mPAP with patients younger than 65 years of age excluded. The correlation between mPAP and age became more correlated than that when excluding old patients (r = −0.2565; p = 0.0043) D. Correlation between age and 6 MWD. The diagram showed a linear trend towards shorter 6 MWD with increasing age (−0.2964, p = 0.001). E. Correlation between age and 6 MWD with patients older than 65 years of age excluded. The trend towards shorter 6 MWD with increasing age became less prominent (r = −0.2129; p = 0.0005) F. Correlation between age and 6 MWD with patients younger than 65 years of age excluded. The correlation between 6 MWD and age turned insignificant (r = −0.1723; p = 0.0986). These diagrams implicated that there might be a homogeneity in the patients younger than 65 years. In addition, patients older than 65 years may actually be suffering from a different kind of PH.

An inverse relationship between age and mPAP was shown in Fig. 5A (r = −0.2715; *p* < 0.001). When patients older than 65 years of age were excluded, the trend towards lower mPAP with increasing age became less prominent (r = −0.1228; *p* = 0.0288; Fig. 5B), compared with the trend in patients older than 65 years old (r = −0.2565; *p* = 0.0043; Fig. 5C).

Moreover, there was a trend towards shorter 6 MWD with increasing age (r = −0.2964; *p* = 0.001; Fig. 5D). When patients older than 65 years of age were excluded, the trend towards shorter 6 MWD with increasing age became less prominent (r = −0.2129; p = 0.0005; Fig. 5E). When the patients younger than 65 years of age were excluded, the correlation between 6 MWD and age turned insignificant (r = −0.1723; *p* = 0.0986; Fig. 5F). Multivariate logistic regression found that age >65 years (OR = 6.23, 95% CI = 2.01–19.31, *p* = 0.0015) and the use of diuretics (OR = 3.46, 95% CI = 1.55–7.74, *p* = 0.0024) were the risks factors for high mortality risk of PH (Table 2).

**Table 2:** Independent risk factors for high risk in ERS/ESC risk stratification model analyzed with multivariate logistic regression.

Variables	OR	95% CI	*p* value
Male sex	0.61	0.28–1.34	0.2205
Age (reference: age ≤ 45yrs)			
Age: 45–65 yrs	1.24	0.58–2.65	0.5827
Age: >65 yrs	6.23	2.01–19.31	**0.0015**
Etiology of PH: group 1 pulmonary arterial hypertension	1.85	0.69–4.99	0.2232
mPAP >25 mmHg	1.47	0.34–6.29	0.6046
BMI >35	1.63	0.38–7.01	0.5095
Heart rate >100 beats/min	3.15	0.98–10.15	0.0546
Systolic blood pressure <90 mmHg	0.52	0.12–2.35	0.3958
Serum creatinine >1.5 mg/dL	1.41	0.24–8.46	0.7059
Hemoglobin <10 g/dL	1.69	0.49–5.81	0.4031
Platelet <100000/μL	1.29	0.29–5.74	0.7378
ALT >40 U/L	0.80	0.28–2.27	0.6724
Use of diuretic drugs	3.46	1.55–7.74	**0.0024**
Treatment (reference: no treatment)			
Monotherapy	0.22	0.01–5.92	0.3639
Duel therapy	0.14	<0.001–65.23	0.5261
Triple therapy	0.38	<0.001–234.29	0.7684

ALT, alanine aminotransferase; BMI, body mass index; CI, confidence interval; mPAP, mean pulmonary arterial pressure; OR, odds ratio; PH, pulmonary hypertension; yrs, years.

## 4 Discussion

The main findings of this analysis include (1) the mean age of patients diagnosed with PH has been increasing compared to previous registries; (2) the distribution of subgroups of PH were different across each age group; (3) there was a different hemodynamic profiles of the elderly patients with PH compared to young patients; (4) there was less proportion of combination therapy in elderly patients; (5) ageing was a significant predictor for high mortality risk predicted by 2015 ESC/ERS risk assessment model for PH patients.

Our study showed that the mean age of PH patients in Taiwan is 55.3, which is comparable to recent studies of registries of PH in Europe, America, and East Asia. A French registry between 2002 and 2003 revealed that the mean age of group 1 PH was 50 [9]. In addition, Registry to Evaluate Early and Long-Term Pulmonary Arterial Hypertension Disease Management (REVEAL) in the United States between 2006 and 2007 demonstrated a mean age of 50.1 years [10]. Furthermore, PH registry in Japan in 2013 showed that mean age of PH patients was 56.0 ^11^. This study confirmed that PH population nowadays is much older than previous reports [4].

As for distribution of subgroups of PH across different age groups, there was significantly lower proportion of group 1 PH in elderly patients compared to young patients, which has been illustrated in several recent registries [6,10,11]. In contrast, our study found a significantly higher proportion of elderly patients were classified as group 4 PH than young patients (Fig. 3), which was supported by previous study [12,13]. Study of Kopeć et al. also reported that 55.4% of patients diagnosed with group 4 PH were older than 65 years were [14]. This may be the result of high prevalence of deep vein thrombosis and pulmonary embolism in the elderly population [15,16]. Similarly, a significantly greater percentage of group 3 PH in elderly patients compared to young patients was reported in our study (Fig. 3). This results were compatible with other registries [12]. Chebib et al. also found that 75.2% of group 3 PH patients were older than 65 years of age [17].

This study denoted an inverse relationship between mPAP and age (Fig. 5A–C). In addition, PVR showed a trend towards lower values with increasing age although the difference of PVR between young patients and elderly patients was not statistically significant (Table 1). Notably, the patients in TAIPANS presented no differences in severity of symptoms. Furthermore, cardiac index, LVEF, and RVEF were similar across three age groups. Exercise capacity did not differ between different age groups except a statistically significantly shorter 6 MWD in elderly patients compared to young patients. Although this reduced 6 MWD may be influenced by aging or comorbidities, it remains a valuable indicator of exercise capacity in patients with pulmonary hypertension. Overall, these findings suggest that elderly patients diagnosed with pulmonary hypertension exhibit poorer exercise capacity despite no significant elevation in mPAP or PVR and similar cardiac index compared to young patients. As COMPERA study suggested, right ventricles of the elderly patients may be weaker and less capable of generating high pulmonary pressure at the same level of PVR as those of young patients [7]. Therefore, elderly patients tended to had more impairment of exercise capacity even though their PVR was comparable with, or lower than (though not statistically significant), that of young patients. A small cohort study in Japan also reported results indicating exercise capacity-hemodynamics mismatch in the elderly group [18].

The higher PAWP in the elderly patient group than in the young patient group raised the possibility that these elderly patients may actually have undiagnosed group 2 PH instead of group 1 PH. Although there was not any patient classified as group 2 PH included in our study, there might be some PH patients with undetected occult LV diastolic dysfunction, which would be disclosed by performing volume or exercise challenge during right heart catheterization. Although there is no consensus as whether to include the fluid or exercise challenge in the diagnostic workup, several studies indicated that these diagnostic procedures were necessary for differentiating group 1 PH to group 2 PH [17,19]. Shapiro et al. demonstrated that PAWP increases after exercise more in patients with PH secondary to severe heart failure with preserved ejection fraction than in those with idiopathic PAH [17]. Furthermore, Robbins et al. found that 22.2% of patients originally classified as group 1 PH exhibited a PCWP >15 mmHg after fluid challenge and were reclassified as group 2 PH [19]. Given the higher PAWP, lower mPAP, and shorter 6 MWD in elderly patients with PH (Table 1), they may be considerably susceptible to occult group 2 PH, or even belonged to a completely new category of PH. Correlation between age and mPAP and correlation between age and 6 MWD (Fig. 5A–F) also indicated the special characteristics of elderly patients with PH. When we excluded patients older than 65 years of age, we noted a weaker correlation between mPAP and age. In addition, correlation between 6 MWD and age became weaker when we excluded patients >65 years. These findings suggested that there might be a homogeneity in the patients younger than 65 years. Moreover, patients older than 65 years may actually be suffering from a different kind of disease.

Regarding medications in PH patients, ERS guideline suggested that initial combination drug therapy should be considered for treatment of group 1 PH [2]. However, there was significantly lower proportion of elderly patients with group 1 PH receiving more than one PH drugs compared to young patients (Fig. 4A) [20,21]. Similar phenomenon was observed in patients with group 3 PH, in which young patients received significantly more off-label dual therapy than the elderly. Registries in UK and Ireland, in Sweden, and COMPERA study all reported similar results [6,7,19]. Nevertheless, limited studies directly investigated the reason why elderly patients less frequently received combination therapy than their young counterparts. One possible explanation for this phenomenon is that a great number of drug interactions between PH-targeted drugs and medications for chronic diseases made it difficult for physicians to prescribe multiple PH-targeted drugs simultaneously for elderly patients who typically presented multiple comorbidities aside from PH [22,23]. Another possible reason for the lack of combination therapy for the elderly could be that the evidence for the efficacy of these targeted medications for the elderly is limited [24,25]. Clinicians may hesitate about administering PH-targeted drugs to the elderly because most clinical trials for PH-targeted drugs excluded elderly patients [26,27]. It is also possible that elderly patients less frequently received combination therapy because they were less capable of tolerating side effects of PH-targeted drugs [21,28]. In COMPERA study [7], more elderly patients discontinued their PH-targeted therapy due to side effects than young patients.

In our multivariate logistic regression, we noted that the use of diuretics and age older than 65 years of age were independent risk factors predicting high mortality risk for PH patients. Most studies focused on the validation of risk stratification model proposed by ESC/ESR in 2015 in mortality rate in the real world [[29], [30], [31]]. On the contrary, our study tried to find out the risk factors associated with high mortality risk based on the strata of 2015 ERS/ESC guidelines [2]. It is reasonable that patients using diuretics have higher mortality risks compared to patients not using diuretics because the necessity of the use of diuretics indicated symptoms of heart failure of higher severity possibly resulting from severe PH. The reason why age >65 years is the risk factors predicting high mortality risk could be that aging and comorbidities of elderly patients made them more fragile and susceptible to PH.

Primary limitation of our study is that we do not acquire follow-up data after the patients were enrolled to our registry. Therefore, we do not possess information regarding survival, treatment response, and the change of treatment over time. Moreover, because most registries enrolled only group 1 PH, it was difficult to make comparison between our study and previous studies; nevertheless, group 1 PH accounted 76.7% of patients in our registry, so it was still reasonable to compared our results with other previous registries. Another limitation of our study is that we established our inclusion criteria based on the 2015 ESC/ESR Guidelines for the diagnosis and treatment of pulmonary hypertension, as our registry commenced in 2019 when the new 2022 guidelines had not yet been published [8,32]. The other limitation of our study is that we could not provide information about the comorbidities of our patients, which may affect our interpretation of the patients’ exercise capacity, hemodynamic profiles, and respiratory parameters. The strength of this study was that it was the first analysis to compare characteristics between elderly patients with PH and their younger counterparts in Asia. We also investigate the predictors for high mortality as to provide more knowledge about the risk assessment and prognosis of PH.

## 5 Conclusion

Our study demonstrated the exercise capacity-hemodynamics mismatch among elderly patients with PH, whose exercise capacity was poorer than young patients despite a lower mPAP. In addition, elderly patients were treated inadequacy, and ageing is a risk factor for high mortality risk. Unique epidemiologic profiles and hemodynamic patterns of elderly patients with PH should be concerned in elderly patients with PH.

## Fundings

This study was supported by grants from 10.13039/501100011913Kaohsiung Veterans General Hospital, Kaohsiung, Taiwan (Grant Nos. KSVGH111-007, KSVGH110-077, VGHKS109-132, VAC110-001-4, and the Ministry of Science and Technology (grant numbers MOST107-2314-B-075B-008-MY2 and MOST108-2314-B-075B-007-MY2).

## Ethics statements

This research was approved by Institutional Review Board in Kaohsiung Veterans General Hospital. The approval number is VGHKS19-CT8-03 TAIPANS.

## Additional information

No additional information is available for this paper.

## CRediT authorship contribution statement

**Chang-Ying Chen:** Writing – original draft, Investigation, Conceptualization. **Wang Mei-Tzu:** Writing – review & editing, Supervision, Investigation. **Shih-Hsien Sung:** Writing – review & editing, Supervision. **Yih-Jer Wu:** Visualization. **Chih-Hsin Hsu:** Supervision. **Wan-Jing Ho:** Data curation. **Yen-Hung Lin:** Formal analysis. **Wei-Shin Liu:** Resources. **Ju-Chi Liu:** Formal analysis. **Yung-Ta Kao:** Formal analysis. **Wen-Shiann Wu:** Visualization. **Chun-Hsien Wu:** Resources. **Meng-Huan Lei:** Data curation. **Yu-Wei Chen:** Data curation. **Chien Chen-Yu:** Resources. **Yu-Wei Chiu:** Funding acquisition. **Zen-Kong Dai:** Funding acquisition. **Tsung-Hsien Lin:** Methodology. **Lin Lin:** Methodology. **Cheng-Chih Chung:** Methodology. **Chang-Min Chung:** Funding acquisition. **Sung-Hao Huang:** Funding acquisition. **Chin-Chang Cheng:** Funding acquisition. **Yen-Wen Wu:** Methodology. **Ting-Hsing Chao:** Supervision, Project administration. **Juey-Jen Hwang:** Resources. **Charles Jia-Yin Hou:** Project administration. **Wei-Chun Huang:** Supervision, Project administration, Methodology, Funding acquisition.

## Declaration of generative AI and AI-assisted technologies in the writing process

During the preparation of this work the author(s) used Microsoft Copilot and Sider in order to enhance the elegance and readability of my research paper. After using this tool/service, the author(s) reviewed and edited the content as needed and take(s) full responsibility for the content of the publication.

## Declaration of competing interest

The authors declare that they have no known competing financial interests or personal relationships that could have appeared to influence the work reported in this paper.
